# Influence of uric acid on the correlation between waist circumference and triglyceride glucose index: an analysis from CHARLS

**DOI:** 10.1186/s12944-021-01474-0

**Published:** 2021-04-30

**Authors:** Jia Zheng, Min Jiang, Yanxia Xie

**Affiliations:** 1 Department of Clinical Epidemiology, the Fourth Affiliated Hospital of China Medical University, Shenyang, 110032 P. R. China; 2 Department of Epidemiology, School of Public Health, China Medical University, Shenyang, 110122 China; 3 National Office for Maternal and Child Health Surveillance of China, Department of Obstetrics, West China Second University Hospital, Sichuan University, Chengdu, 610041 P. R. China

**Keywords:** Waist circumference, Uric acid, Triglyceride glucose, Temporal relationship

## Abstract

**Background:**

Waist circumference (WC) and uric acid (UA) are significantly related. Still, their temporal sequence and how the sequence works on future risk of triglyceride glucose (TyG) are unknown, especially in the Chinese population.

**Methods:**

Cross-lagged panel model was used to analyze the reciprocal, longitudinal relationships among a set of interrelated variables. The mediation model was constructed to test the effect of the relationship between WC and UA on TyG.

**Results:**

A total of 5727 subjects were enrolled in our study population, of which 53.5% were women, and the mean age was 59.0 (standard deviation, 8.62) years. After adjusting for traditional confounding factors, the results showed that a higher level of baseline WC was significantly associated with a higher level of follow-up UA (*β* = 0.003, *P* = 0.031) and follow-up TyG (*β* = 0.003, *P <* 0.001);. Simultaneously, there was no statistical association between the level of baseline UA and the level of follow-up WC (*β* = − 0.009, *P =* 0.951). The mediation effects of UA on WC-TyG were estimated to be 18.1% in adults, and 36.2% in women.

**Conclusions:**

The current study demonstrated that higher baseline level of WC probably preceded UA’ level in general population. In addition, UA mediated the relationship of WC to TyG, especially in females. And the possible mechanism would require further clarification.

## Background

Insulin resistance (IR) [1–5] and uric acid (UA) [6–11] are closely associated with some noncommunicable diseases (NCDs), including cardiovascular disease (CVD), metabolic syndrome (MS), type 2 diabetes mellitus (T2DM), all-Cause and cause-specific mortality. Therefore, early detection and control of IR and UA are necessary to reduce the incidence risk of NCDs, especially among the high-risk, symptom free populations.

Recent researches have reported that visceral fat (determined by waist circumference [WC]) is related to IR [12–14], and some studies have found that WC is a simpler and more effective indicator used to discriminate IR than other indicators, such as waist to height ratio [15]. Growing evidence demonstrates that abdominal adiposity, evaluated by WC, causes more health risks than total adiposity, evaluated by body mass index (BMI) [16]. In addition, some researches have indicated that the measurement of obesity was positively correlated to serum uric acid (UA) [17–19]. And on one Mendelian Randomization study relating UA as an early metabolic disorder having upstream effect in development of more traditional risk factors and CVD itself [20]. Some studies have found that increased of serum UA is related to the increased risk of IR-based clinical diseases and the relationship related to gender [8, 21, 22]. The level of serum UA is also related with the increase of body fat deposition [23, 24].

TyG levels, which are associated with IR, play significant roles in predicting the incidence of diabetes and CVD. However, it is unclear whether WC is related to TyG and to what possible extent. To date, there have been few general population studies, particularly prospective cohort studies of Chinese adults who have increased WC and TyG. At the same time, a large American population study showed that the inverse association between serum antioxidant levels and inflammatory markers is moderated by adiposity [25]. On this basic, the possible association of WC and TyG mediated by UA was hypothesized in the current study. Therefore, the current research aimed to explore the relationship between WC and UA to TyG level. In addition, a separate subgroup analysis was conducted to evaluate the differential effects of WC, UA, and TyG between male and female.

## Methods

### Study participants

China Health and Retirement Longitudinal Study (CHARLS) was a representative survey research nationwide conducted by the National School of Development of Peking University. Between June 2011 and March 2012 (baseline survey), a multistage process with random cluster sampling was conducted to select a representative sample of individuals older than 45 years in 10,287 households in 450 villages/cities. 17,708 individuals were enrolled in the baseline survey through face-to-face household interviews. After the initial recruitment, all 17,708 individuals have been re-surveyed every two years using the same questionnaire as at the baseline. Blood samples were gathered in the year of 2011 and 2015. The detailed information regarding the CHARLS has been described on the website of CHARLS: http://charls.pku.edu.cn/en. The study protocol has been approved by ethical committees of Peking University (IRB 00001052–11,014), and all individuals have provided written informed consent.

**Fig. 1:**
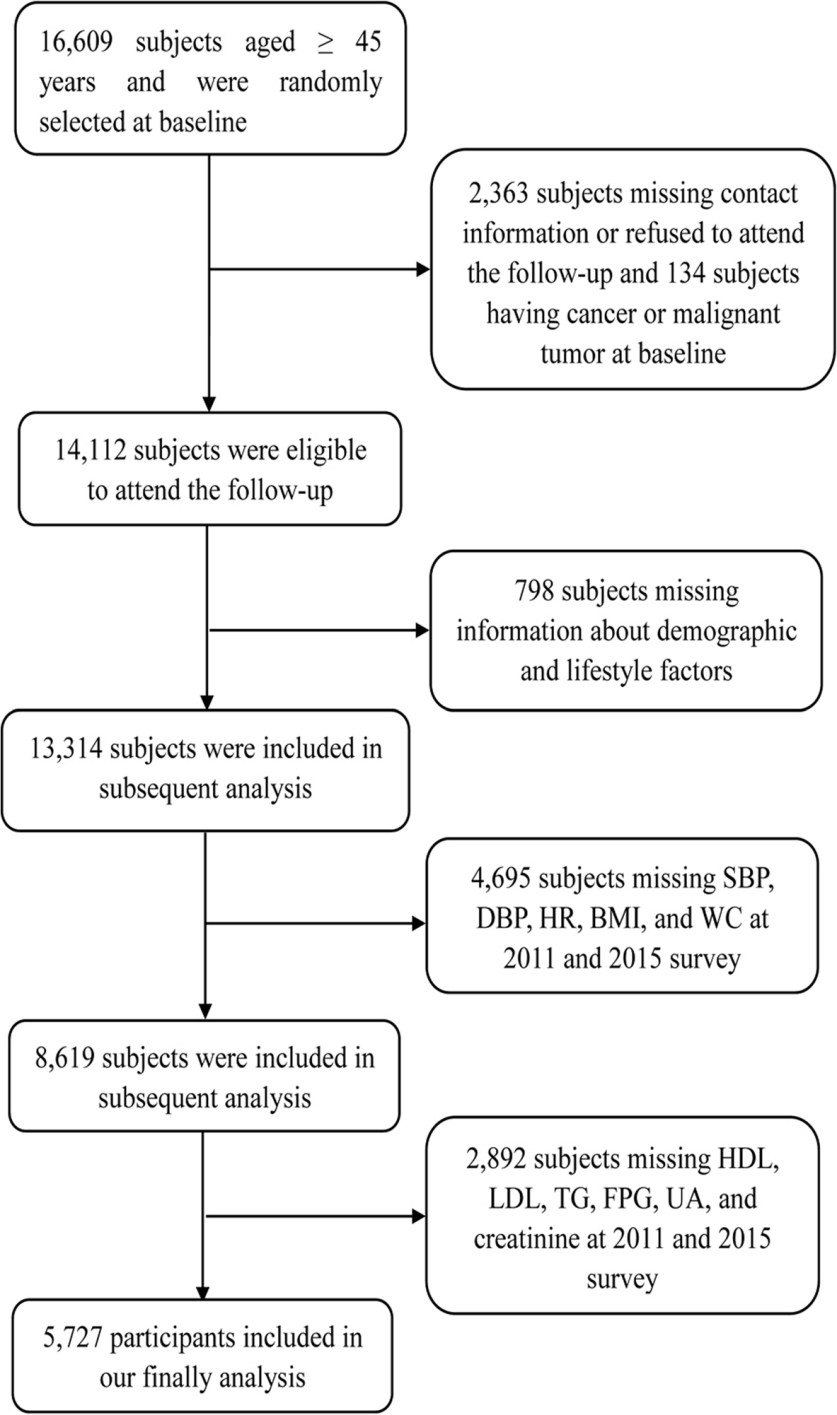
Flowchart of participants included in this study after inclusion and exclusion

This study was a post hoc analysis of CHARLS from 2011 to 2015. Of the 17,708 individuals at baseline, 1099 subjects were younger than 45 years. Participants with detailed follow-up information, major study variables and confounding variables at baseline and follow-up period were included in the study. Participants who had cancer at baseline were excluded. Finally, 5727 subjects were included in our study. The flowchart in Fig. 1 was used to show the detailed information about the sample size of the subjects and the exclusion criteria in the current research.

### Exposures and covariates

Information on participants demographics (gender, age, ethnicity, and education), lifestyle factors (smoking status and drinking status), and history of diseases (diabetes, chronic kidney disease, and hypertension) were collected during the structured household survey. Data on healthy behaviors were obtained from the subjects’ self-reported questionnaire, including smoking status (former, current smoker or never), frequency of alcohol consumption (more than once a month, once a month or never). Data on diabetes, chronic kidney disease, and hypertension were collected by trained health staff members.

Data on collection and measurement of cholesterol indexes, fasting plasma glucose, biochemical blood indexes, and other blood pressure indexes are detailed on the website of CHARLS: http://charls.pku.edu.cn/en. The mean of 3 blood pressure values was calculated and used for our analysis. The BMI was calculated using the weight and height indicators; the formula as follows: BMI = weight (Kg) / height^2^ (m^2^).

### Dependent variable (Y)

The TyG index was calculated according to the following formula: TyG = ln [fasting TG (mmol/L) × FPG (mmol/L) × 0.5 × 159.37]. TG and FPG were measured by enzymatic colorimetric test method.

### Independent variable (X)

A soft tape was inserted at the navel level to measure WC in a standing pose with a cloth measuring tape. At the same time, all participants needed to do a regular breathing exercise, holding the breath at the end of exhaling and letting the tape out slightly.

### Mediators (M)

UA plus method was used to measure UA.

### Statistical analysis

All analyses were conducted by IBM SPSS version 22.0 and SPSS Amos 22.0 with *P* ≤ 0.05 (two-sided) considered statistically significant differences.

Percentiles were calculated used to describe the categorical variables, while arithmetic mean with standard deviation (SD) was calculated for the description of continuous variables that met the normal distribution. The median with interquartile range was used to describe the continuous variables that didn’t meet the normal distribution. At the same time, the Student t test, the Mann-Whitney U test or the Pearson’s χ2 test was used for the comparison between male and female.

The linear regression model was constructed to explore whether WC (UA) could predict the future variation of TyG. Future TyG variation and baseline WC (UA) were the dependent and independent variables of the model, respectively. Firstly, the multicollinearity problem among independent variables was examined by the variance expansion factor (VIF). VIF greater than 10 was deemed significant multicollinearity. Secondly, three models were constructed to assess the relationship between baseline (examination at 2011) WC (UA) and future (follow-up at 2015) TyG as follows: model 1: adjusted for baseline WC (UA); model 2: model 1 plus sex, ethnicity and age; model 3: model 2 plus drinking, current smoking, education, BMI, LDL-C, HDL-C, HR, DBP, SBP, and creatinine.

**Fig. 2:**
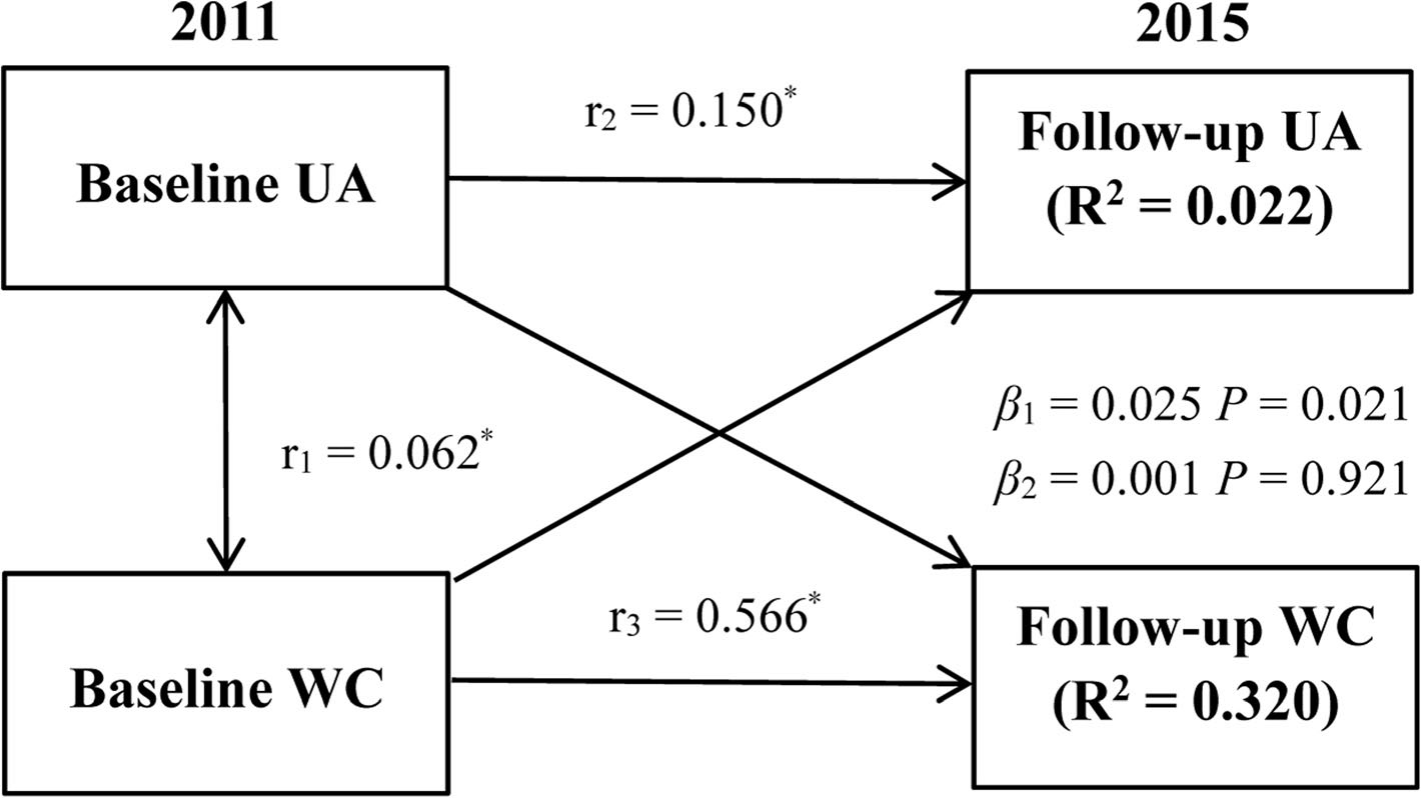
Cross-lagged path analysis of WC and UA index(*n* = 5727), adjusted for baseline and follow-up variables, respectively: sex, age, ethnicity, education, current smoking, alcohol consumption. r_1_ = synchronous correlations; r_2_, r_3_ = tracking correlations; r_1_, r_2_ = cross-lagged path coefficients; R^2^ = variance explained; * represent coefficients different from 0, *P* < 0.001; CFI = 0.991, RMR = 0.016

Longitudinal changes of WC and UA indexes were measured at two follow-up periods. Previous study introduced the theory and application of cross-lagged panel design [26]. Overall, the cross-lagged panel model was performed for the longitudinal relationships among different interrelated variables [27]. In Fig. 2, the path with *β*_1_ showed the impact of baseline UA on follow-up WC, and *β*_2_ showed the effect of baseline WC on follow-up UA. WC and UA values were adjusted in linear regression analysis by baseline and follow-up variables: sex, age, ethnicity, alcohol consumption, current smoking, education, BMI, LDL-C, HDL-C, HR, DBP, SBP, and creatinine. Residuals were saved and after that, Z-transformation was used to standardize the saved residuals (mean = 0; SD = 1). Root-mean-square residual (RMR) and comparative Fit Index (CFI) and were enrolled for the model fits. RMR < 0.05 and CFI > 90 meant a relatively good model fit in the cross-lagged path model. In addition, this analysis model was constructed in groups of men and women, separately.

**Fig. 3:**
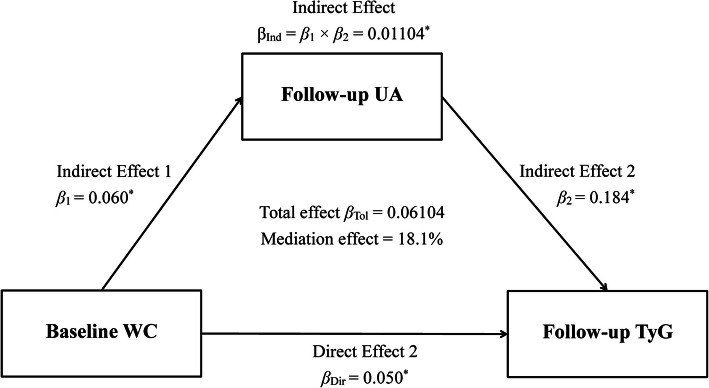
Mediation effect of UA index on the WC-TyG association (n = 5727), * represent coefficients different from 0, *P* < 0.001

When the temporal relationship was establised between WC and UA, the mediation model would be fitted to explore the potential influence of the association between UA and WC on TyG. The values of TyG were analyzed by linear regression residual model and standardized by Z-transformation (with mean of 0 and SD of 1). According to the results of the cross-lagged path analysis model, X and M were determined as predictor and mediator, respectively. The detailed mediation model was showed in Fig. 3, which included three models, given by:

$$ \mathrm{Model}\ \mathrm{Y}={\beta}_{\mathrm{Tol}}\ \mathrm{X}\kern0.5em \left({\beta}_{\mathrm{Tol}}=\mathrm{total}\ \mathrm{effect}\right), $$

$$ \mathrm{Model}\ \mathrm{M}={\beta}_1\ \mathrm{X}\ \left({\beta}_1=\mathrm{indirect}\ \mathrm{effect}\ 1\right), $$

$$ \mathrm{Model}\ \mathrm{Y}={\beta}_2\ \mathrm{M}+{\beta}_{\mathrm{Dir}}\ \mathrm{X}\ \left({\beta}_2=\mathrm{indirect}\ \mathrm{effect}\ 2,{\beta}_{\mathrm{Dir}}=\mathrm{direct}\ \mathrm{effect}\right). $$

The following formula was used to calculate the proportion of the mediation effect, as follows: Mediation effect (%) = $\frac{\beta_1\times {\beta}_2}{\beta_{\mathrm{Tol}}}\times 100\%$.

## Results

In the end, the study enrolled 5727 participants, of which 53.5% was female. The mean age of this population was 59.0 ± 8.62 years. Detailed baseline and follow-up characteristics of the study population are summarized in Table 1. By comparing the difference between sex groups, the results indicated that HR, DBP, SBP, and FPG were not significantly different at baseline and FPG was not significantly different at follow-up.

**Table 1:** Baseline Characteristics of Study Participants by sex and abdominal obesity (n = 5727)

Characteristic	Men (n = 2663)	Women (n = 3064)	*P*	Total
**Baseline (2011)**				
Age (years)	59.9 (8.57)	58.2 (8.58)	< 0.001	59.0 (8.62)
Ethnicity, n (%)				
Han	2491 (93.5)	2820 (92.0)	0.029	5311 (92.7)
Others	172 (6.5)	244 (8.0)		416 (7.3)
Education, n (%)				
Illiteracy	852 (32.0)	1853 (60.5)	< 0.001	2705 (47.2)
Primary school	764 (28.7)	572 (18.7)		1336 (23.3)
Middle school	709 (26.6)	456 (14.9)		1165 (20.3)
High school	222 (8.3)	146 (4.8)		368 (6.4)
Tertiary high school or above	116 (4.4)	37 (1.2)		153 (2.7)
Current smoking, n (%)	2010 (75.5)	240 (7.8)	< 0.001	2250 (39.3)
Drinking ≥1 times/month, n (%)	1214 (45.6)	225 (7.3)	< 0.001	452 (7.9)
SBP (mmHg)	129.7 (19.96)	130.0 (21.59)	0.537	129.8 (20.85)
DBP (mmHg)	75.9 (12.34)	75.4 (11.70)	0.121	75.6 (12.00)
HR (beats/min)	71.9 (10.79)	72.4 (9.70)	0.051	72.2 (10.22)
HDL-C (mg/dL)	48.33 (39.05–59.54)	50.26 (41.37–60.31)	< 0.001	49.48 (40.21–59.92)
LDL-C (mg/dL)	109.41 (89.30–131.44)	118.30 (96.26–142.66)	< 0.001	114.05 (93.17–137.24)
BMI (Kg/m2)	23.0 (3.56)	24.1 (3.94)	< 0.001	23.6 (3.81)
Waist Circumference (cm)	84.1 (11.83)	84.8 (12.81)	0.044	84.5 (12.37)
Uric Acid (mg/dL)	4.88 (1.233)	3.98 (1.042)	< 0.001	4.40 (1.221)
Creatinine (mg/dL)	0.87 (0.189)	0.69 (0.136)	< 0.001	0.77 (0.186)
FPG (mmol/L)	6.10 (1.931)	6.12 (1.925)	0.686	6.11 (1.928)
TG (mmmol/L)	1.43 (1.147)	1.55 (1.025)	< 0.001	1.50 (1.085)
TyG*	6.33 (0.669)	6.44 (0.643)	< 0.001	6.39 (0.658)
**Follow-up (2015)**				
Current smoking, n (%)	1392 (52.3)	167 (5.5)	< 0.001	1559 (27.2)
Drinking ≥1 times/month, n (%)	1187 (44.6)	243 (7.9)	< 0.001	1430 (25.0)
SBP (mmHg)	130.5 (19.73)	129.4 (20.23)	0.032	129.9 (20.00)
DBP (mmHg)	76.3 (11.35)	74.7 (10.89)	< 0.001	75.4 (11.13)
HR*	73.3 (11.19)	74.3 (10.19)	0.001	73.8 (10.68)
HDL-C	48.26 (41.70–56.70)	51.35 (44.79–58.69)	< 0.001	50.19 (43.24–57.92)
LDL-C	96.52 (79.54–115.06)	105.79 (86.49–123.94)	< 0.001	101.54 (83.01–120.08)
BMI (Kg/m2)	23.2 (3.73)	24.3 (4.24)	< 0.001	23.8 (4.05)
Waist Circumference (cm)	84.3 (13.80)	85.7 (13.41)	< 0.001	85.0 (13.61)
Uric Acid (mg/dL)	5.46 (1.419)	4.48 (1.215)	< 0.001	4.94 (1.403)
Creatinine (mg/dL)	0.92 (0.304)	0.72 (0.207)	< 0.001	0.81 (0.275)
FPG (mmol/L)	5.75 (1.997)	5.82 (2.022)	0.207	5.79 (2.010)
TG (mmmol/L)	1.51 (0.996)	1.73 (1.044)	< 0.001	1.62 (1.028)
TyG	6.34 (0.643)	6.50 (0.627)	< 0.001	6.43 (0.640)

*Abbreviations*: *UA* uric acid, *WC* waist circumference, *TyG* triglyceride-glucose, *SBP* systolic blood pressure, *DBP* diastolic blood pressure, *HR* heart rate, *BMI* body mass index, *HDL-C* high-density lipoprotein cholesterol, *LDL-C* low-density lipoprotein cholesterol, *TG* Triglycerides, *FPG* fasting blood glucose

TyG = ln [fasting TG (mmol/L) * FPG (mmol/L) *0.5*159.37]

BMI = Weight (kg)/height (m)^2^

Table 2 shows the prospective correlation of baseline WC and follow-up UA and TyG. In model 3, the result revealed that a higher value of WC was related with a higher follow-up UA value(*β* = 0.003, *P* = 0.031) and follow-up TyG level (*β* = 0.003, *P <* 0.001).

**Table 2:** Prospective associations of baseline WC with follow-up UA and TyG

	Follow-up UA		Follow-up TyG	
	*β* (95%CI)	*P*	*β* (95%CI)	*P*
Modle 1				
WC	0.007 (0.005–0.009)	< 0.001	0.014 (0.013–0.015)	< 0.001
Modle 2				
WC	0.008 (0.006–0.010)	< 0.001	0.013 (0.012–0.014)	< 0.001
Modle 3				
WC	0.003 (0.000–0.006)	0.031	0.005 (0.003–0.006)	< 0.001

Model 1: adjusted for baseline UA

Model 2: adjusted for factors in model 1 and baseline age, ethnicity and sex

Model 3: adjusted for all variables in model 2 plus baseline education, current smoking, alcohol drinking, SBP, DBP, HR, BMI, HDL-C, LDL-C, creatinine

*Abbreviations*: *UA* uric acid, *WC*, waist circumference, *TyG* triglyceride-glucose, *SBP* systolic blood pressure, *DBP* diastolic blood pressure, *HR* heart rate, *BMI* body mass index, *HDL-C* high-density lipoprotein cholesterol, *LDL-C* low-density lipoprotein cholesterol

Table 3 showed the prospective relationship of baseline UA with follow-up WC and TyG. The results indicated that a higher baseline UA value was related to a higher follow-up TyG value(*β* = 0.051, *P <* 0.001). However, there was no significant association between baseline UA and follow-up WC (*β* = − 0.009, *P =* 0.951).

**Table 3:** Prospective associations of baseline UA with follow-up WC and TyG

	Follow-up WC	*P*	Follow-up TyG	*P*
Modle 1				
UA	0.221(−0.033–0.474)	0.088	0.037 (0.023–0.050)	< 0.001
Modle 2				
UA	0.552 (0.279–0.826)	< 0.001	0.074 (0.060–0.088)	< 0.001
Modle 3				
UA	−0.009(− 0.284–0.267)	0.951	0.051 (0.037–0.066)	< 0.001

Model 1: adjusted for baseline WC

Model 2: adjusted for factors in model 1 and baseline age, ethnicity and sex

Model 3: adjusted for all variables in model 2 plus baseline education, current smoking, alcohol drinking, SBP, DBP, HR, BMI, HDL-C, LDL-C, creatinine

*Abbreviations*: *UA* uric acid, *WC* waist circumference, *TyG* triglyceride-glucose, *SBP* systolic blood pressure, *DBP* diastolic blood pressure, *HR* heart rate, *BMI* body mass index, *HDL-C* high-density lipoprotein cholesterol, *LDL-C* low-density lipoprotein cholesterol

Results of the cross-lagged path analysis for WC and UA in the general population with age over 45 are shown in Fig. 2. The WC_baseline_ → UA_follow-up_ path coefficient is 0.025 (*P* = 0.021). However, the UA_baseline_ → WC_follow-up_ path coefficient is 0.001 (*P* = 0.921). The study results showed that a wider WC at baseline resulted in increased UA levels at follow-up, but an increased baseline UA level did not lead to a wider WC. The values of the model fitting parameters in the WC with UA model (CFI = 0.991 and RMR = 0.016) showed a relatively good fit to the observed data in our cross-lagged path analysis.

Figure 3 shows the mediation effects of follow-up TyG on the relationship between baseline WC and follow-up UA, adjusted for sex, age, ethnicity, alcohol drinking, current smoking, education, BMI, LDL-C, HDL-C, HR, DBP, SBP, and creatinine, after excluding cancer at baseline. The standardized total and direct effect of WC on TyG was 0.06104 and 0.050, respectively. According to the formula in the statistical analysis section, the pooled indirect effect was 0.01104 (0.060 × 0.184), while the percentage of the total effects mediated by follow-up UA was 18.1% ($\frac{0.01104}{0.06104}\times 100\%$).

**Fig. 4:**
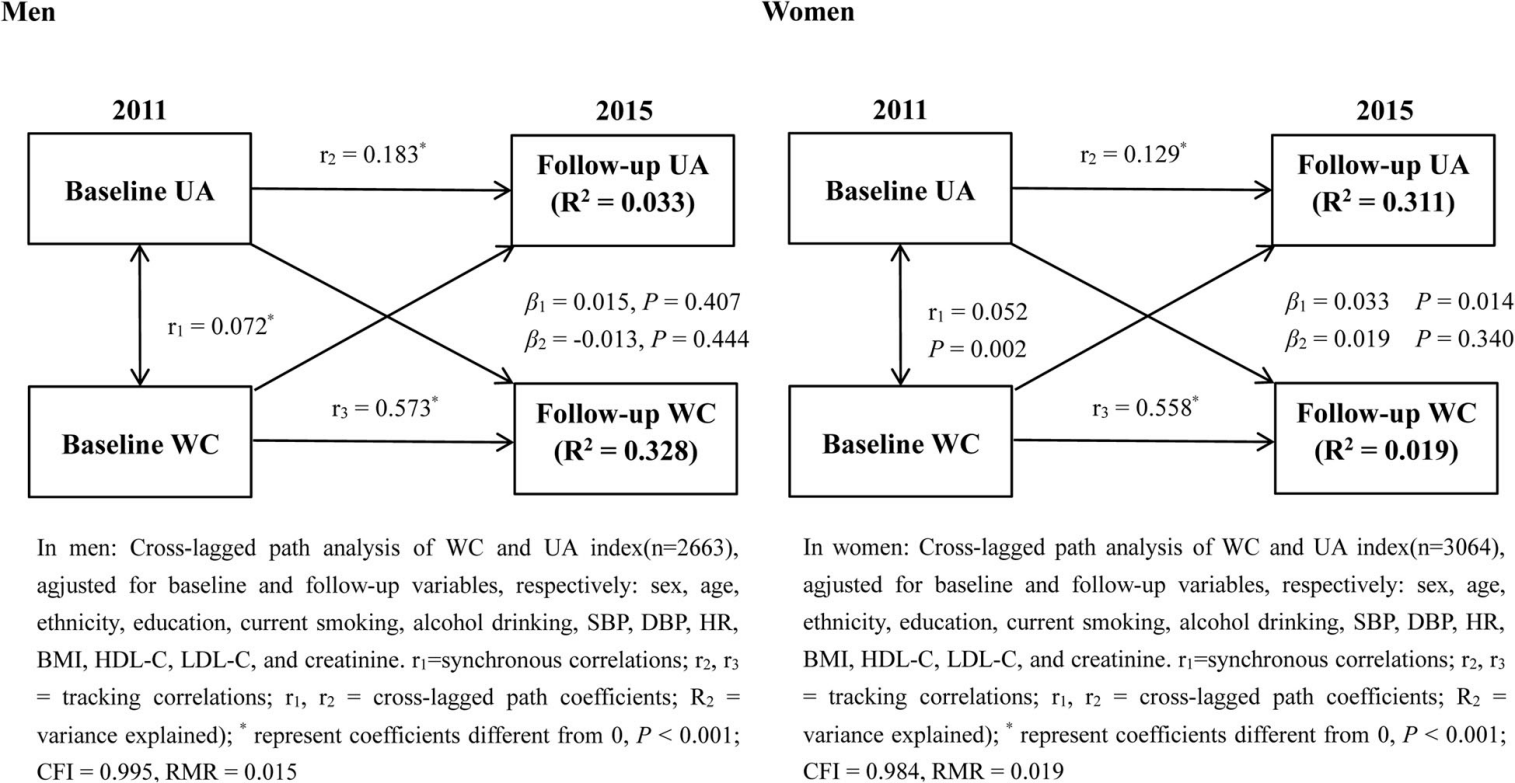
In men: Cross-lagged path analysis of WC and UA index(*n* = 2663), adjusted for baseline and follow-up variables, respectively: sex, age, ethnicity, education, current smoking, alcohol consumption, SBP, DBP, HR, BMI, HDL-C, LDL-C, and creatinine. r_1_ = synchronous correlations; r_2_, r_3_ = tracking correlations; r_1_, r_2_ = cross-lagged path coefficients; R^2^ = variance explained; * represent coefficients different from 0, *P* < 0.001; CFI = 0.995, RMR = 0.015; In women: Cross-lagged path analysis of WC and UA index (*n* = 3064), adjusted for baseline and follow-up variables, respectively: sex, age, ethnicity, education, current smoking, alcohol consumption, SBP, DBP, HR, BMI, HDL-C, LDL-C, and creatinine. r_1_ = synchronous correlations; r_2_, r_3_ = tracking correlations; r_1_, r_2_ = cross-lagged path coefficients; R^2^ = variance explained; * represent coefficients different from 0, *P* < 0.001; CFI = 0.984, RMR = 0.019

**Fig. 5:**
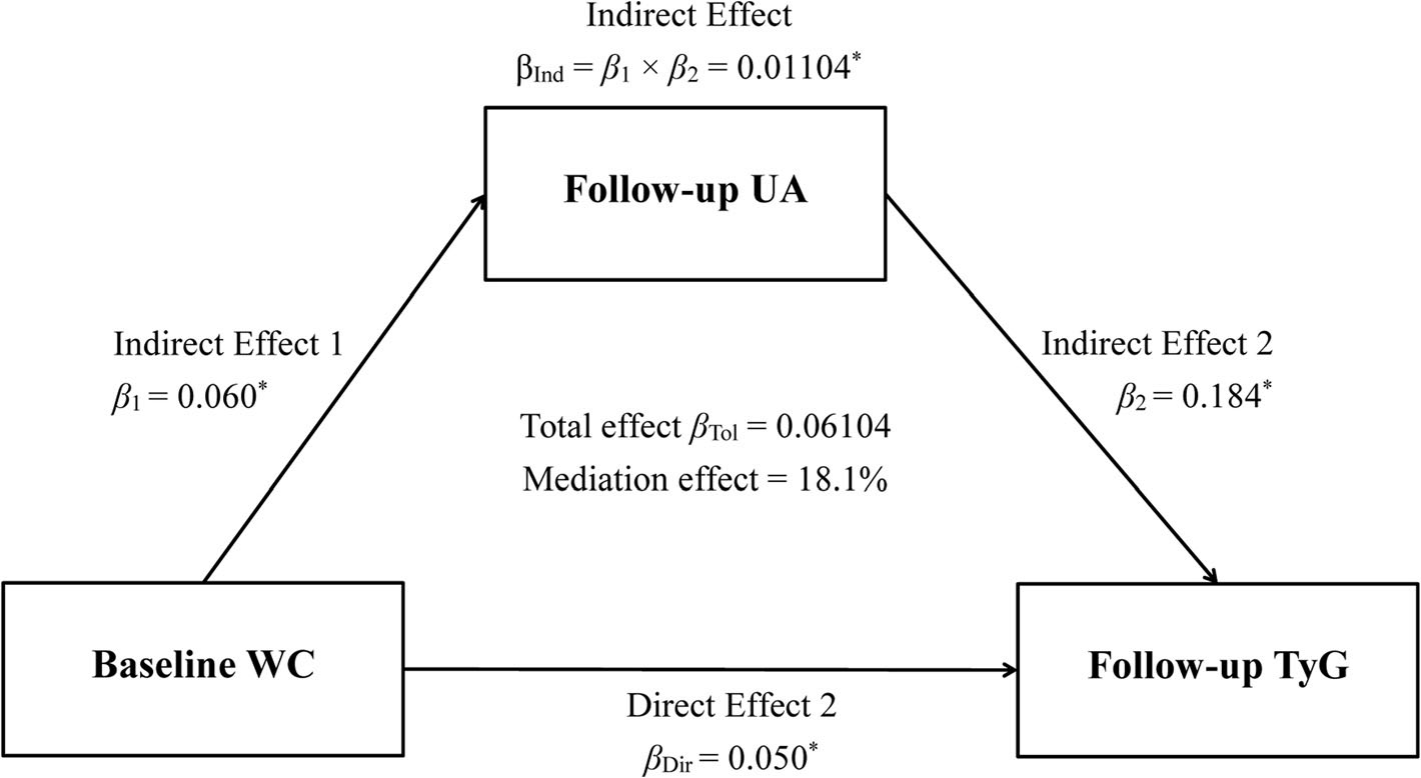
Mediation effect of UA index on the WC-TyG association (n = 3064) in women, * represent coefficients different from 0, *P* < 0.001

The results of subgroup analyses between men and women are shown in Figs. 4 and 5. For women, the results showed that the WC_baseline_ → UA_follow-up_ path coefficient is 0.033 (*P* = 0.014), and the UA_baseline_ → WC_follow-up_ path coefficient is 0.019 (*P* = 0.340). However, for men, we found that the WC_baseline_ → UA_follow-up_ path coefficient is 0.015 (*P* = 0.407), and the UA_baseline_ → WC_follow-up_ path coefficient is − 0.013 (*P* = 0.444). These results indicated that an increase in baseline WC might affect a subsequent UA increase, but only in women, not in men. For women, the percentage of the overall effect mediated by follow-up UA was estimated at 36.2% ($\frac{0.01362}{0.03762}\times 100\%$).

## Discussion

Results of current research were 4-fold: (1) clarifying the temporal relationship between WC and UA (WC_baseline_ → UA_follow-up)_; (2) verifying UA mediate effect on the relationship between WC and TyG; (3) estimating the percentages of UA mediation effect on WC - TyG (18.1%); (4) and finding the mediation effect of UA on WC - TyG in women not in men.

It is widely known that with bad dietary habits and sedentary lifestyle, together with the increase of social pressure. Furthermore, the global obesity rate has shown apparent upward trend [1, 2]. Studies have shown a strong relationship between obesity and disease, and abnormal fat distribution in the body, especially the accumulation of abdominal fat, has a higher correlation with the occurrence of IR [12–14]. WC is a simple and reliable measure of central obesity. Studies have demonstrated that WC can be used as an indicator to assess early insulin secretion [15]. In addition, serum UA and obesity are related to an elevated level of IR and the incidence of MS [21]. Other studies have demonstrated that UA was an independent risk factor for gout and some major public health threats, such as CVD, MS, and even stroke [6–10]. WC as the obesity indicator is considered the main risk factor for hyperuricemia [23, 24], however, the temporal relationship between WC and hyperuricemia is still controversial. In this study, the cross-lagged path analysis was applied to explore the chicken-and-egg question because it is a practical statistical approach to analyze the causal relationship between two intercorrelated variables. In current study, the results revealed a unidirectional relationship from WC to UA. In addition, because sex may influence the level of WC, UA, and TyG, a subgroup analysis was conduced in male and female, separately. Unidirectional relationship from WC to UA was significantly stronger in female than that in male. UA mediated the effect of WC on TyG, and the mediation effect of UA was only observed in women, not in men. The reason for gender differences needs in-depth studies.

A previous study has shown that half of diabetes cases could be attributed to the increased serum UA level [28]. The increased level of serum UA is known to be related to hypertension, glucose intolerance, and obesity [29]. A previous study demonstrated that the high level of UA predicted the development of hyperinsulinemia [30]. Therefore, the mediation impact of UA on IR should be considered when examining the association of WC and IR. The high level of UA was positively correlated with the expression level of high-sensitivity C-reactive protein [31]. Oxidative stress in adipose tissue is a risk factor for IR because in oxidative stress insulin sensitivity decreases in adipose tissue. Simultaneously, soluble UA might lead to increased generation of reactive oxygen species (ROS) and elevated tissue NADPH oxidase levels. Increasing serum UA can cause IR by reducing the bioavailability of nitric oxide and lead to oxidative stress in mitochondria. In 2014 Viazzi et al. [32] stated that this mechanism happens because of hyperinsulinemia that acts as an excellent marker in measuring IR. In addition, another two researches have demonstrated that the association between UA and CVD outcomes was U-shaped curve [33, 34]. And this may be the potential reason that this mechanism may also exist in the case of low UA concentrations.

The rising IR-related health burden emphasizes the significance and urgency for searching and managing risk factors for IR. Therefore, early detection and control of IR is crucial, and it should be carried out even in the absence of symptoms. Recently, homeostatic model assessment (HOMA-IR) has been commonly used as measurement index. However, the HOMA-IR is time-consuming and expensive. Some studies have developed and assessed surrogate markers for IR [35]. One of these biomarkers is the TyG, and it has been examined in different populations with stable results, although these studies were carried out in normal-weight adults [36]. Therefore, the TyG was used in the research.

Prevalence of abdominal obesity has increased and has been considered a major health hazard, which is significantly correlated with the risk of mortality, CVD, as well as other diseases. Comparing to generalized obesity, central obesity is more closely linked to IR. WC as a predicted measure of health risk is a convenient and effective index of abdominal obesity. It has a significant, predictable association with intra-abdominal fat area and volume. One cross-sectional study of participants without diabetes demonstrated that the insulin sensitivity index significantly correlated with WC across populations [36]. Previous literature evidence has found that WC is a suitable proxy measure for central obesity [15]. Studies have shown that with the increase of BMI and adipose tissue, the incidence of T2DM gradually increases [16]. However, the ratio of adipose tissue to muscle will affect the BMI, so the situation of human adipose tissue cannot be entirely and objectively reflected. Additionally, increased triglyceride-rich lipoprotein secretion and impaired clearance of these lipoproteins are the potential mechanisms for central obesity and hypertriglyceridemia [37, 38]. After controlling for BMI, WC as a marker of abdominal obesity, has been significantly associated with T2DM risk [39]. In addition, it has been debated that CVD and IR are associated with the increase in visceral adipose tissue, and WC is a better marker of changes in fat distribution comparing to BMI.

The Finnish Diabetes Prevention Study with impaired glucose tolerance risk population showed that the change in UA was significantly related to the change in fasting insulin [40]. The serum level of UA as the purine metabolism, is determined by the level of purine metabolism in the liver, excretion from the kidney, and secretion from the adipose tissue. Some studies proved that central obesity might be associated with hypertriglyceridemia by increasing the triglyceride-rich lipoprotein secretion and impairing this lipoprotein clearance [37, 38]. The mechanism may include the abnormal secretion of very-low-density lipoproteins associated with the increased visceral adiposity [37].

### Study strength and limitations

All WC, UA, and TyG measurements were carried out by trained staff using unified and standardized measuring instrument with high repeatability. Most importantly, we conducted the subgroup analysis between males and females about the UA’s mediation effects on WC to IR in a representative sample of the Chinese population with age ≥ 45 years. The study added to the understanding of the relationship between UA and IR in the context of obesity and metabolic syndrome. Additionally, the current study provided information on alcohol consumption, current smoking, education, BMI, LDL-C, HDL-C, HR, DBP, SBP, and creatinine. After excluding the missing data, only 5727 participants were included in our finally analysis.

## Conclusions

The results of this research demonstrated that the baseline level of WC and UA was associated with the future risk of IR. Furthermore, the temporal relationship between WC and UA was WC_baseline_ → UA_follow-up_, and the mediation effect of UA on WC - TyG was found in women, but not in men. Therefore, early WC detection might be helpful for the primary care physicians to stratify the potential patients that need the further blood screening. Further researches are required to explore the possible underlying mechanism.

## Data Availability

All the data can be found on the CHARLS website (http://charls.pku.edu.cn/en). The datasets analyzed during current study are available from the corresponding author on reasonable request.

## Abbreviations

IR: Insulin resistance
UA: Uric acid
NCDs: Noncommunicable diseases
T2DM: Type 2 diabetes mellitus
MS: Metabolic syndrome
CVD: Cardiovascular disease
WC: Waist circumference
BMI: Body mass index
CHARLS: China Health and Retirement Longitudinal Study
SD: Standard deviation
VIF: Variance expansion factor
CFI: Comparative fit index
RMR: Root-mean-square residual
